# Driving Frequency and Well-Being in Older Adulthood

**DOI:** 10.1093/geroni/igab046.2777

**Published:** 2021-12-17

**Authors:** Zainab Suntai, Kefentse Kubanga, Abhay Lidbe, Emmanuel Adanu

**Affiliations:** University of Alabama, Tuscaloosa, Alabama, United States

## Abstract

The activity theory of aging suggests that older adults age successfully when they remain active and engaged. While many older adults are still able to drive, not all are as engaged in social activities, despite having the transportation to be able to do so. As such, this study aimed to examine the association between the frequency of driving and overall well-being among older adults. The hypothesis is that older adults who drive more frequently would have higher well-being, as they are likely driving to engaging activities. A sample of 1,663 older adults who reported that they are able to drive were derived from the 2018 National Health and Aging Trends Study (NHATS). The NHATS is an annual longitudinal panel of survey of adults aged 65 and older living in the United States. Chi-square tests were used for bivariate analyses and a weighted multivariable logistic regression model was used to predict well-being based on driving frequency. Results showed that compared to those who drive every day, those who drive most days (OR=0.771, CI= [0.768-0.775]), some days (OR=0.495, CI= [0.492-0.497]), rarely (OR=0.558, CI= [0.555-0.562]) or never (OR=0.371, CI= [0.367-0.374]) were less likely to have high well-being. Interventions geared at improving well-being among older adults should therefore consider increasing awareness of social events, to ensure that older adults who are able to drive can have a good quality of life by driving to social activities.

